# Supplementary material for: Deep Learning Convolutional Neural Network Reconstruction and Radial k-Space Acquisition MR Technique for Enhanced Detection of Retropatellar Cartilage Lesions of the Knee Joint
Source: Diagnostics (Basel). 2023 Jul 21;13(14):2438. doi: 10.3390/diagnostics13142438 (PMC10378433; doi:10.3390/diagnostics13142438)
Supplement: Supplementary file 1 [file diagnostics-13-02438-s001.zip › diagnostics-2427512-supplementary.pdf]

Table S1. Database search table.

**Database**	**Pubmed**			
**Search Keywords**	"deep learning AND propeller AND MRI"			
**Results**	Title	Authors	Journal	Year
1	Deep Learning and Imaging f or the Orthopaedic Surgeon: How Machines "Read" Radiographs.	Hill BG, Krogue JD, Jevsevar DS, Schilling PL.	J Bone Joint Surg Am.	2022
2	Comparison of deep learning-based reconstruction of PROPELLER Shoulder M RI with conventional reconstruction	Hahn S, Yi J, Lee HJ, Lee Y, Lee J, Wang X, Fung M	Skeletal Radiol.	2023
3	Impact of Deep Learning Reconstruct ion Combined With a Sharpening Filter on Single-Shot Fast Spin-Echo T2-Weighted Magnetic Resonanc e Imaging of the Uterus.	Tsuboyama T, Onishi H, Nakamoto A, Ogawa K, Koyama Y, Tarewaki H, Tomiyama N.	Invest Radiol.	2022
4	Scientific Advances and Technical Innovations in Musculoskeletal Radiology.	Fritz J, Runge VM.	Invest Radiol.	2023
5	Application of deep learning-based image reconstruction in MR imaging of the shoulder joint to improve image quality and reduce scan time.	Kaniewska M, Deininger-Czermak E, Getzmann JM, Wang X, Lohezic M, Guggenberger R.	Eur Radiol.	2023
6	A Feasibility Study on Deep Learning Reconstruc tion to Improve Image Quality With PROPELLER Acquisiti on in the Setting of T2-Weighted Gynecologic Pelvic Magnetic Resonance I maging.	Saleh M, Virarkar M, Javadi S, Mathew M, Vulasala SSR, Son JB, Sun J, Bayram E, Wang X, Ma J, Szklaruk J, Bhosale P.	J Comput Assist Tomogr.	2023
**Database**	**Pubmed**			
**Search Keywords**	"deep learning AND propeller AND MRI AND knee"			
**Results**	"No results were found."			
**Database**	**Scopus**			

**Search Keywords**	"deep learning AND propeller AND MRI"			
**Results**	Title	Authors	Journal	Details
1	Comparison of deep learning-based reconstruction of PROPELLER Shoulder MRI with conventional reconstruction	Hahn, S., Yi, J.,Lee, H.-J., .Wang, X., Fung, M.	Skeletal Radiology	52(8), pp. 1545–1555
2	Application of deep learning–based image reconstruction in MR imaging of the shoulder joint to improve image quality and reduce scan time	Kaniewska, M., Deininger-Czermak, E., Getzmann, J.M., .Lohezic, M., Guggenberger, R.	European Radiology	33(3), pp. 1513–1525
3	Breast Diffusion MRI Acquisition and Processing Techniques: The GE Healthcare Perspective	Shimakawa, A., Bayram, E.	Diffusion MRI of the Breast	pp. 251–25
4	The Reconstruction Method Using Compressed Sensing and Convolutional Neural Network for PROPELLER MRI in Head	Matsumoto, Y., Hori, K., Tadano, K., .Endo, Y., Hashimoto, T.	2021 IEEE Nuclear Science Symposium and Medical Imaging Conference Record	NSS/MIC 2021 and 28th International Symposium on Room-Temperature Semiconductor Detectors, RTSD 2022,
**Database**	**Scopus**			
**Search Keywords**	"deep learning AND propeller AND MRI AND knee"			
**Results**	"No documents matching your keywords were found."			
**Database**	**Web of Science**			
**Search Keywords**	"deep learning AND propeller AND MRI"			
**Results**	Title	Authors	Journal	Details
1	Comparison of deep learning-based reconstruction of PROPELLER Shoulder MRI with conventional reconstruction	Hahn, S; Yi, J; (...); Fung, M	SKELETAL RADIOLOGY	52 (8), pp.1545-1555

2	Application of deep learning-based image reconstruction in MR imaging of the shoulder joint to improve image quality and reduce scan time	Kaniewska, M; Deininger-Czermak, E; (...); Guggenberger, R	EUROPEAN RADIOLOGY	33 (3) , pp.1513-1525
3	Impact of Deep Learning Reconstruction Combined With a Sharpening Filter on Single-Shot Fast Spin-Echo T2-Weighted Magnetic Resonance Imaging of the Uterus	Tsuboyama, T; Onishi, H; (...); Tomiyama, N	INVESTIGATIVE RADIOLOGY	57 (6) , pp.379-386
4	Dual-domain self-supervised learning for accelerated non-Cartesian MRI reconstruction	Zhou, B; Schlemper, J; (...); Sofka, M	MEDICAL IMAGE ANALYSIS	81
5	Comprehensive Clinical Evaluation of a Deep Learning-Accelerated, Single-Breath-Hold Abdominal HASTE at 1.5 T and 3 T	Herrmann, J; Wessling, D; (...); Othman, AE	ACADEMIC RADIOLOGY	30 (1) , pp.93-102
6	Pediatric brain extraction from T2-weighted MR images using 3D dual frame U-net and human connectome database	Kim, D; Chae, JH and Han, Y	INTERNATIONAL JOURNAL OF IMAGING SYSTEMS AND TECHNOLOGY	29 (4) , pp.476-482
7	Stochastic optimization of three-dimensional non-Cartesian sampling trajectory	Wang, GH; Nielsen, JF; (...); Noll, DC	Magn Reson Med	Apr 2023 (Early Access)
8	Motion artifact reduction for magnetic resonance imaging with deep learning and k-space analysis	Cui, L; Song, Y; (...); Yang, G	PLOS ONE	18 (1)
**Database**	**Web of Science**			
**Search Keywords**	"deep learning AND propeller AND MRI AND knee"			
**Results**	"No records were found to match your filter"			
